# Medicinal Effect, *In Silico* Bioactivity Prediction, and Pharmaceutical Formulation of *Ageratum conyzoides* L.: A Review

**DOI:** 10.1155/2020/6420909

**Published:** 2020-10-13

**Authors:** Jasvidianto C. Kotta, Agatha B. S. Lestari, Damiana S. Candrasari, Maywan Hariono

**Affiliations:** Faculty of Pharmacy, Sanata Dharma University, Yogyakarta Campus III, Depok 55282, Indonesia

## Abstract

Goat weed (*Ageratum conyzoides* L.), or bandotan in Indonesia, is an herbaceous plant that broadly grows up in both subtropical as well as tropical areas. This herb contains many phytoconstituents which have many benefits in different aspects. The essential oil contains phytochemicals such as phenol, phenolic ester, and coumarin, whereas many compounds can been identified in the whole part such as terpenoid, steroid, chromene, pyrrolizidine alkaloid, and flavonoid. Empirically, this herb has been used as an antihemorrhagic, antiseptic, antileprosy, and wound-healing agent. This article reviews the potency of the herb in medication according to the chemical substances being deposited, which are collected from numerous studies, followed by its *in silico* bioactivity prediction as well as its pharmaceutical dosage form formulation.

## 1. Introduction

**Figure 1:**
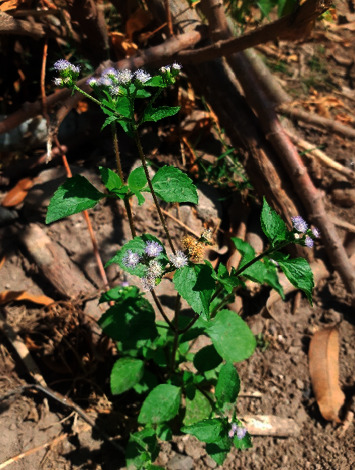
Habitus of *Ageratum conyzoides L.*

The utilization of plants as traditional medicine has been directly changing a paradigm from the use of a synthetic drug to a natural product (back to nature). *Ageratum conyzoides* L., namely, bandotan in Indonesia, is an herb that grows up broadly in both subtropical and tropical areas. *Ageratum* was named from Ancient Greek “*a geras*” means stay young and *conyzoides* derived from “*konyz*” means plants. This herb is genuinely from tropical America having a goat-like smell and hence called “*goat weed*” in English [1]. *Ageratum conyzoides* L. is a family of Compositae with *Ageratum* as the genus. Most of this genus is categorized as an herb due to its height with at least 30 species. This herb has stems and leaves covered by smooth white trichomes alongside the leaves having a short petiole. Furthermore, the leaf blade has an egg-like circular shape and 7.5 cm in length which is sharpening up to its terminal. The flowers consist of 8 to 15 receptacles in either purple or white color, whereas the fruits are black and small. This herb is easily found in from rice fields, yard, jungle, roadside, and riverside having a lot of sun exposure [2].  Figure 1 is the habitus of *Ageratum conyzoides* L. photographed in Maguwoharjo, Specific Region of Yogyakarta, Indonesia.

*Ageratum conyzoides* L. is an annual herb with a long history in its benefits as a traditional medicine in many countries in the world. This weed has been known since the past time for its medicinal effect in various diseases such as common wound and the burned one, antimicrobe, arthrosis, headache, and dyspnea. The further medicinal effect has been reported such as antipneumonia, pain killer, anti-inflammatory agent, antiasthma, antispasmodic, haemostatic, gastrointestinal disorder, gynaecological disorder, antileprosy, and many other skin diseases [3].

In Africa, goat weed has been used traditionally to relieve constipation and fever and applied as a wound dressing agent as well as an antiulcer agent. In Togo, this herb is used to treat measles and snake bite, whereas in Nigeria, it is used for skin diseases, wound healing, diarrhoea, pain around the navel in children, and even used to treat HIV/AIDS [4]. The leaves are consumed as vegetables and also to prevent tetanus [5]. It is also used in the medication of pneumonia, antitoxin of snake venom, typhoid fever, malaria fever, sore throat, and candidiasis. The roots are used in the treatment of tumours, lithiasis, and diarrhoea in a baby. Moreover, the flower is used to relieve itching, insomnia, cough, vermifuge, tonics, and antibug parasite [6]. Besides its benefits in medication, this weed is also utilized in agriculture as an organic material which can increase a soil nutrient composition. A study evaluating this weed administration in the bokashi preparation toward the cultivation and the nutrient composition of a tomato plant demonstrates a significant effect in a mass of the tomato fruits [7].

*Ageratum conyzoides* L. has abroad pharmacological activities, in which one study reported that its ethanolic extract had antiurolithiatic activity which can prevent kidneys from urinary stone formation [8]. They also possess pharmacological activities such as analgesics and antipyretics [9, 10], anti-inflammation [10–12], antinociceptive [13], antioxidant [11, 14], cancer cytotoxic [15], antiulcerogenic [16, 17], antidiabetes [18–20], anticataleptic [21], antimicrobe [22, 23], antitumor and anticancer [24, 25], hepatoprotective [26, 27], anticonvulsant [28], radioprotective [29], anticoccidial [30], antidote [31], antiprotozoal [32], hematopoietic [33], allelopathic [34], bronchodilator dan uterine relaxant [35], anthelmintic [36], insecticide [37], anti-Ehrlichia [38], wound healing [39], gastroprotective [40], and anti-HIV/AIDS [41].

**Figure 2:**
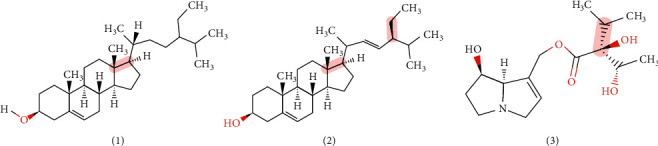
The molecule structure of the main constituents of *Ageratum conyzoides* L.

*Ageratum conyzoides* L. contains phytochemical substances such as terpenoid, sterol, flavonoid, chromene, pyrrolizidine alkaloid, coumarin, pyrrolon, and lignan [5]. Other studies also reported its essential oil phytoconstituent includes kaempferol, rhamnoside, quercetin, scutellarein, chromene, stigma-7-*en*-3-ol, sitosterol, stigmasterol, fumaric acid, caffeic acid, saponin, pyrrolidine alkaloid, ageratochromene derivatives, and alkane [6]. The main constituents (see Figure 2) are *β*-sitosterol (1), stigmasterol (2) [42], and lycopsamine (3) in which (1) and (2) are class of sterol, whereas (3) is pyrrolizidine alkaloid class [43].

A recent study has reported that *Ageratum conyzoides* L. leaves extract containing quercetin actively inhibited TNF-*α* during the inflammation process by degrading the collagenase of cartilage as well as the matrix metalloproteinase-9 (MMP-9). TNF-*α* is a cytokine inflammatory agent involved in osteoarthritis, which can be worsening inflammatory status in a blood vessel wall by inducing the endothelial cell damage, regulating the leucocyte activity, and releasing the cytokine as well as chemokine proinflammatory agent [44]. MMP-9 is a zinc-dependent peptidase, a member of MMP superfamily [45]. MMP is classified into five major groups according to their substrate specificity which is collagenase (MMP-1, 8, and 13), gelatinase (MMP-2 and 9), stromelysin (MMP-3, 10, 11, and 19), matrilysin (MMP-7, 12, and 18), and membrane-bound MMP (MMP-1 and 4) [46, 47].

*Ageratum conyzoides* L. contains phytochemicals with various potencies in medication, particularly in the drug discovery and development from herbal. It is also interesting to explore the pharmacological effect as well as its pharmaceutical formulation. This article reviews the active phytoconstituents of this herb which are responsible for its pharmacological activities followed by an *in silico* prediction of the bioactivity and the pharmaceutical preparations that have been formulated up to now.

## 2. Pharmacological Activities

### 2.1. Analgesic

The analgesic activity has been reported for this herb by using alcoholic extract of a whole part of the plant [10]. *In vitro* study demonstrated that *Ageratum conyzoides* L. performed analgesic activity in the ovarium of the Chinese hamster [48]. Further study by Dewan et al. also supported that the crude ethanolic extract of its leaves exhibited analgesic activity in mice induced by acetic acid [49]. During inflammation, the elevation of prostaglandin levels affects poor pain by enhancing the capillary permeability [50]. At this stage, the synthesis of prostaglandin is inhibited through the mechanism of peripheral pain inhibition leading to its analgesic effect [51]. A clinical study has been carried out in patients with arthritis using a water extract from the whole part of the *Ageratum*. The result shows that 66% of the patients are relieved from their pain, and their articulation mobility was improved by 24% without side effects [52].

**Figure 3:**
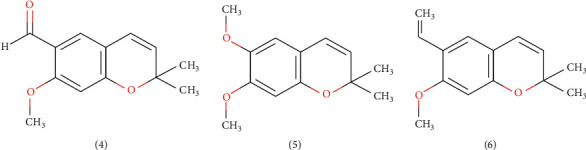
Molecule structure of chromene which are responsible for the analgesic activity of *Ageratum conyzoides* L.

A study supported the previous study by exhibiting a significant analgesic activity of water extract from the whole part of *Ageratum conyzoides* L. using the tail flick method [9]. The phytochemicals of *Ageratum conyzoides* L. essential oils employing precocene I (4), precocene II (5), and 6-vinyl-7-methoxy-2,2-dimethyl chromene (VMDC) (6) (see Figure 3) are a member of chromene compounds, responsible for its analgesic bioactivity [53]. Furthermore, the alkaloid and flavonoid in the oil can block the pain perception [54], whereas other secondary metabolites such as tannin, saponin, and terpenoid also have been reported having pharmacological activity [55].

### 2.2. Anti-Inflammatory Agent

**Figure 4:**
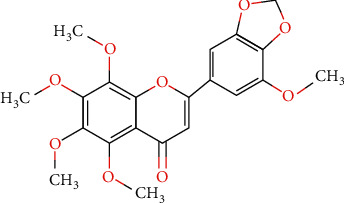
Molecule structure of 5′-methoxynobiletin which is a benefit for its anti-inflammatory effects of *Ageratum conyzoides* L.

The ethanolic extract of ageratum can reduce inflammation significantly, which is induced by carrageenan and histamine activities. The phytochemical substances playing a role in this activity are quercetin, kaempferol, glycoside, tannin, and other polyphenolic compounds [56]. The potential anti-inflammatory agent from this herb due to the inflammation was induced by raw oil which is further characterized by DNA damage in a male Wistar mouse sperm [57]. *In vitro* study of this methanolic extract also showed anti-inflammatory activity, which supports the previous study [58]. A study related to this standardized herbal extract by 5′-methoxynobiletin which is a class of polymethoxyflavones (see Figure 4) showed some benefits to overcome pain and inflammation. This provides a new innovation in the development of a new drug to treat inflammation-related diseases such as rheumatoid arthritis [13].

One study reported that the anti-inflammatory effect of goat weed depended on the flavonoid fraction which influenced the protein expression from the inflammatory gen [11]. In particular, the flavonoid glycoside fraction of this herb showed a very potent anti-inflammatory activity [12]. In a mouse pleural cavity, the inflammatory response is reduced by administrating this herb's extract, by blocking the entry of leucocyte and the protein exudate concentration, and also diminishing the level of the inflammatory mediator [59].

### 2.3. Antimicrobial Effect

The antimicrobial activity in both methanol and ether extract of *Ageratum conyzoides* L. has been reported. This herb contains antimicrobial components which are useful against oxidase enzyme in bacteria which is a pathogen and related to the systemic infection, leading to deadly effect in animal and human [22]. Another study also explores that essential oil in *Ageratum conyzoides* L. had a potential antimicrobial towards *Escherichia coli* and *Klebsiella pneumoniae* (uropathogenic bacteria) and, therefore, is useful in the prophylactic of urinary tract infection [60].

The antimicrobial activity assay from both root and leaf extracts demonstrates antibacterial and antifungal activities against the isolate of *Bacillus subtilis* (Gram positive), *Escherichia coli*, *Klebsiella pneumoniae*, *Pseudomonas aeruginosa*, *Staphylococcus aureus*, *Salmonella typhi* (Gram negative), and fungal strain, i.e., *Aspergillus niger*, *Candida albicans*, *Penicillium notatum*, and *Rhizopus stolon* [61]. The essential oil of *Ageratum conyzoides* L. leaves has antibacterial activity in inhibiting *Salmonella enteritidis* [62]. The leaves and stem extract of *Ageratum conyzoides* L. can be used as a broad-spectrum antibacterial agent. Moreover, the *in vitro* study from the methanolic extract and *n*-hexane extract of *Ageratum conyzoides* L. showed antifungal activities against *Fusarium solani* [63]. The leaves and stem extracts of *Ageratum conyzoides* L. contain phytochemical substances such as flavonoid, saponin, alkaloid, tannin, and phenol, which showed antibacterial activity depends on its concentration against the bacterial isolate [64].

### 2.4. Antioxidant

The ethanolic extract of leaves and flowers from *Ageratum conyzoides* L. demonstrated antioxidant activities against DPPH (1,1-diphenyl-2-picryl hydrazyl) to scavenge the free radicals. Those antioxidant activities are due to the presence of phytochemicals such as flavonoids and phenol [65]. This claim was supported by the study of the potent antioxidant from methanolic extract of *Ageratum conyzoides* L. stems depending on the phenolic components [15]. The result shows that the leaves of the ethanolic extract have antioxidant activity which is higher than its water extract. This extract has a strong natural antioxidant effect against the free radical and also used in the treatment of erectile dysfunction induced by oxidative stress [14].

Further study about the antioxidant activity from the leaves ethanolic extract has also supported the previous study [66]. On the one hand, the methanolic extract shows much higher antioxidant activity than its essential oils [67]. On the other hand, *Ageratum conyzoides* L. has a positive effect in the redox system when it is exposed to diabetic rats induced by streptozotocin, while increasing the glycaemic status of those rats [68].

### 2.5. Antimalaria

A study shows that the water extract of *Ageratum conyzoides* L. has a potency to increase the antimalaria activity of chloroquine and artesunate in the plasmodial induced-rats [69]. The phytochemical responsible for the antimalarial activities are alkaloid, glycoside, flavonoid, saponin, tannin, and resin. In this study, the leaves extract as well as its fraction show a significant antimalarial activity toward parasite plasmodium as confirmed by *in vitro* or *in vivo* model in mice [69].

### 2.6. Agent for Metabolic and Degenerative Diseases

Ethanolic extract of *Ageratum conyzoides* L. demonstrates antidiarrhoea and antidiabetic activities in albino rats [19]. The water extract also showed activity as hypoglycemic in diabetic rats induced by normoglycemic and streptozotocin [20]. Other studies also investigated the antidiabetic activity of the leaves water extract by exhibiting a positive effect on hypoglycemic activities [70]. The extract of goat weed has a hypoglycemic effect which is significantly comparable to glibenclamide [71]. Another study also reported that *Ageratum conyzoides* L. demonstrates both hypoglycemic as well as hypolipidemic effects in rats [72].

*Ageratum conyzoides* L has reported having antidiarrhoea activity through its antispasmodic activity and the solid formation of the feces mass. The ethanolic extract has potency as the gastroprotective agent in rats which could be mediated by its antioxidant properties, by blocking the calcium channel obstruction and its antiserotonergic properties [40].

## 3. Toxicological Activities

Hydroalcoholic extract of *Ageratum conyzoides* L. could be toxic to the liver, kidneys, and blood cells after long-term exposure as confirmed by both *in vivo* and *in vitro* assay [4]. Therefore, the use of *Ageratum conyzoides* L. in a longer duration should be carefully managed. This toxic effect could be due to the alkaloid total especially to pyrrolizidine. The hepatotoxic effect after oral administration of the leaves ethanolic extract has been shown in Wistar albino rats upon three regimen doses, i.e., 200, 400, and 600 mg/kg of bodyweight within 21 days. The experiment shows that the total protein serum and liver alanine aminotransferase (ALT), aspartate aminotransferase (AST), and alkaline phosphatase (ALP) did not significantly change; therefore, it can be concluded that the extract is not toxic to the liver at those respective doses [73].

A study related to the toxicological extract of the leaves has been carried out in rats by showing LD_50_ extract equal to 600 mg/kg bodyweight. The postmortem report shows an alteration in the intestine liver and kidneys, bleeding at subcontracted muscles alongside intestinal tracts, paracentral hepatic necrosis, and tubulin proximal convulsion. However, in the chronic toxicity study, the extract did not show a significant effect in the bodyweight elevation, haematology, its alanine amino serum, the aspartate aminotransferase, and its blood nitrogen urea. This however depends on the dose which affected the histopathological intestine liver and kidneys [74].

## 4. *In Silico* Prediction of Molecular Pharmacology Mechanism

The living organism is graded in seven levels according to their sizes from the smallest to the biggest as follows: ionic, atomic, molecular, cellular, tissue, organ, and body [75]. In the past, the pharmacological activity of the drug was mainly examined using animal experiments [76]. However, due to the ethical clearance, the use of animal in a preliminary study which means at organ and body levels has been tightly restricted [77]. The technology of cell culture and tissue engineering then significantly overcomes the ethical issue; however, at the tissue and cellular levels, there are still a lot of pathways of pathogenesis which made the mechanism become more complicated [78]. Later on, modern drug discovery has been utilizing protein, signal transduction, genes, and nucleic acid as the therapeutic target at the molecular level. This leads to a more selected target because the drug-target interaction can be elucidated specifically [79]. The advanced bioinformatics alongside information technology facilitated the study of drug-target interaction at the molecular level using the *in silico* (computational) method before applying the *in vitro* experiment [80].

This section will predict the interactions of seven representative compounds of *Ageratum conyzoides* with seven proteins from respected biological activities as described before.  Table 1 organizes seven compounds which could affect the corresponding protein target and also corresponds to the diseases of interest. Therefore, this prediction could inform on how *Ageratum conyzoides* works against pathologic conditions at the molecular level. The *in silico* method being used is molecular docking as supplemented in Supplementary Materials (S1). The seven compounds (ligands) are precocene I, precocene II, VMDC, beta-sitosterol, stigmasterol, polymethoxyflavone, and pyrrolizidine, whereas the seven proteins are human tyrosinase (5m8s) [81], *Plasmodium falciparum* ornithine delta-aminotransferase (3lg0) [82], *Mus musculus* cyclooxygenase-2 (4cox) [83], *E. coli* 4-diphosphodicytidyl-2-C-methylerythritol synthase (1i52) [84], glycoside hydrolase family 97 from *Bacteroides thetaiotaomicron* (2zq0) [85], human p53 (4ibu) [86], and human MMP-9 (1l6j) [87].

Tyrosinase is an enzyme associating with the oxidation process due to its function in melanin or other pigment production from tyrosine. A compound that can inhibit tyrosinase could be related to its antioxidant properties [88]. Ornithine delta-aminotransferase has a function to produce nonessential amino acid proline from ornithine which is involved in the plasmodium life cycle that leads to malaria disease [89]. Cyclooxygenase-2 catalyses the conversion of arachidonic acid into inflammatory mediators such as prostaglandin, prostacyclin, and leukotriene leading to inflammation [90]. 4-Diphosphodicytidyl-2-C-methylerythritol synthase is an enzyme expressed by the bacteria cell of *E. coli* having a function to catalyse the formation of isoprenoid which is one of the components in bacteria building block [91]. Glycoside hydrolase family 97 hydrolyses the glycosidic bond between oligosaccharide and polysaccharide into monosaccharide leading to blood glucose upregulation associating with diabetes [92]. p53 is a gene suppressing a tumour gene by upregulating apoptosis. This gene is often associated with cancer cell proliferation [93]. MMP-9 is gelatinase having a function as introduced before, related to inflammation, wound healing, and cancer metastasis [94–96].

Table 2 tabulates the molecular docking results performing binding energy of seven ligands identified in *Ageratum conyzoides* in which the lower energy (more negative) is the stronger binding with the respected protein. In human tyrosinase (5m8s), the chromene compound class (precocene I, precocene II, and VMDC) has better interactions than other compounds, whereas the sterol compound class predominates the better interactions with ornithine delta-aminotransferase than chromene, flavonoid, and alkaloid. In cyclooxygenase-2, either sterol of flavonoid has a better interaction than others, whereas flavonoid is the best ligand to interact with 4-diphosphodicytidyl-2-C-methylerythritol synthase. In contrast, the chromene and alkaloids are even better in interacting with glycoside hydrolase than sterol and flavonoid. Compared with other proteins, all ligands have relatively poor interactions with the p53 gene, whereas the opportunity of *Ageratum conyzoides* as an MMP-9 inhibitor could be due to the presence of sterol and flavonoid than alkaloid and chromene.

**Figure 5:**
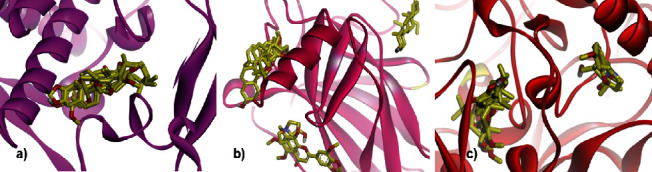
Binding pose of seven ligands identified from *Ageratum conyzoides* in the pocket of (a) human tyrosinase, (b) human p53, and (c) glycoside hydrolase. The protein was presented in a ribbon model, whereas the ligands were in a stick form.

Figure 5 visualizes the binding pose of seven ligands as the docking results in the respected proteins/gene. Although the binding energy is within the broad range, all ligands occupied the same pocket of human tyrosinase. In contrast, the ligands are divided into three binding clusters in the pocket of the human p53 gene. As happened in human p53, the ligands have two different binding positions in the pocket of glycoside hydrolase.

**Figure 6:**
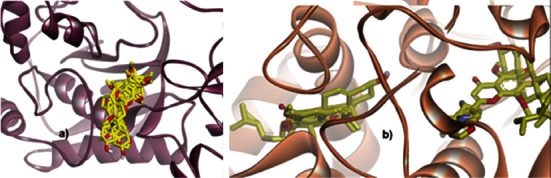
Binding pose of seven ligands identified from *Ageratum conyzoides* in the pocket of (a) MMP-9 and (b) cyclooxygenase-2. The protein was presented in a ribbon model, whereas the ligands were in a stick form.

The binding pose of all ligands is quite stable in the MMP-9 pocket site along with their binding energies. In contrast, the ligands are divided into two binding site positions when they are docked into the cyclooxygenase-2 pocket. Interestingly, two ligands, i.e., beta-sitosterol and polymethoxyflavone (5-methoxynobiletin) performed two lowest binding energies (–8.9 and –8.5 kcal/mol, respectively) when they interacted with cox-2. This means that both ligands bind to the cox-2 binding site stronger than other ligands. Although both ligands have a similar low binding energy, however, their binding positions are different which could lead to a different biological activity (see Figure 6).

**Figure 7:**
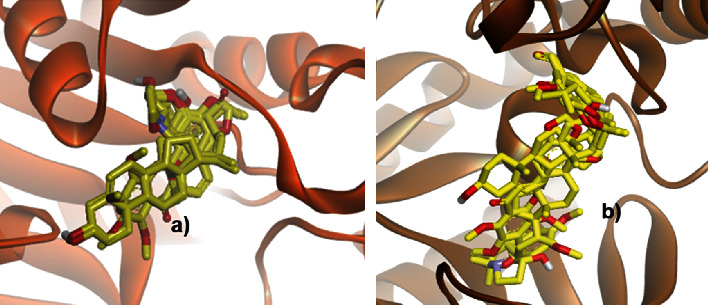
Binding pose of seven ligands identified from *Ageratum conyzoides* in the pocket of (a) 4-diphosphodicytidyl-2-C-methylerythritol synthase and (b) ornithine delta-aminotransferase. The protein was presented in a ribbon model, whereas the ligands were in a stick form.

In microorganisms such as *E. coli* and *P. falciparum*, the ligands are occupying the same binding pocket of 4-diphosphodicytidyl-2-C-methylerythritol synthase and ornithine delta-aminotransferase, respectively. Moreover, the chance of *Ageratum conyzoides* to be antibacterial and antimalarial agents is opened due to the relatively lower binding energies as well as its stable conformations during interaction with the corresponding proteins (see Figure 7).

## 5. Pharmaceutical Formulation

The formulation utilizing extract from goat weed has been carried out. Extract of goat weed could be developed into a polyherbal formulation as the wound-healing agent through a topical route. This formulation is composed of *Ageratum conyzoides*, *Ficus religiosa*, *Curcuma longa*, and *Tamarindus indica*. The result showed that the polyherbal formulation has a good wound-healing effect in rats [97].

The next study also developed the formulation of *emulsifiable concentrate* (EC) from the aerial part extract of *Ageratum conyzoides* L. using a nontoxic solvent and emulsifying agent. This study was focused on the evaluation of such formulations as an antimicrobial agent. A diverse solvent has been used to extract the materials, including dichloromethane, methanol, and *n*-hexane. The identified compounds in the extract elucidated using gas chromatography-mass spectroscopy (GC-MS) showing bioactivities. The developed EC was found more active than its crude extract against bacteria and fungus [98].

The success of EC formulation is developed from the aerial part making further formulation from this herb to be designed in a pharmaceutical gel dosage form. This dosage form then tested its activity against *Staphylococcus epidermidis* and *Propionibacterium acnes*. In this work, *Ageratum conyzoides* was extracted using the maceration method in ethanol 90%. The antibacterial activity was determined using the disc paper diffusion method, whereas the *minimum inhibitory concentration* (MIC) and *minimum bactericidal concentration* (MBC) were determined using the microdilution method. The extract was formulated into the gel basis containing various concentrations of sodium carboxymethyl cellulose (CMC) and hydroxypropyl methylcellulose (HPMC). The result showed that the dosage form performed antibacterial activity against *Staphylococcus epidermidis* and *Propionibacterium acnes* with a MIC value of 2.5%. The best result was performed by the formula containing 4% of the sodium CMC and 2.5% of the extract. The antibacterial activity of the gel was able to inhibit 14.7 ± 2.3 mm and 15.43 ± 1.6 mm in the zone of the growth of *Staphylococcus epidermidis* and *Propionibacterium acnes*, respectively [99].

A study related to the gel formulation was also developed by Taufid and Ameilia. In this study, the extract was prepared by macerating *Ageratum conyzoides* L. using ethanol 96%. The gel was composed of sodium CMC as the bases using three different extract concentrations, i.e., 2.5%, 5%, and 7.5%, accordingly. The physical quality of the gel was evaluated employing physical appearance, homogeneity, pH, and its dispersion force. The gel met the requirement as a good dosage form, in particular with its dispersion force within 3–5 cm [100].

A current study has developed a novel formulation which is nanoemulgel from *Ageratum conyzoides* L. extract (ACE) combined with *Oldenlandia corymbosa L* extract (OCE), indicated as an anti-inflammatory agent. Nanoemulgel ACE-OCE has been developed either individually or in combination, having a good physical characteristic and promising effect from its activity as an anti-inflammatory agent for osteoarthritic therapy [101].  Table 3 organizes the type of extracts that have been formulated in pharmaceutical dosage forms regarding their corresponding active ingredient being studied previously for their molecular interaction in the protein targets of interest.

## 6. Discussion

The aerial part of *Ageratum conyzoides* has been elaborated above, as this plant has the potential to be developed as a modern drug. The identified compound has extensively studied its pharmacological activity as a pain killer, anti-inflammatory, antioxidant, antimalarial, antidiabetes, anticancer, and antibacterial agent. The most-reported compounds being studied, having a responsibility toward its pharmacological activity, are classes of chromene, sterol, flavonoid, and alkaloid.

The chromene compounds studied as an analgesic drug are corresponding with its *in silico* prediction in which precocene I, precocene II, and VMDC performed relatively good energy bindings in cox-2. Furthermore, polymethoxyflavone studied as anti-inflammatory agents is exhibiting better binding energy with cox-2 than chromene and other ligands. Flavonoids such as polymethoxyflavone also were studied as an antibacterial against *E. coli* which could be due to the 4-diphosphodicytidyl-2-C-methylerythritol synthase inhibition. In contrast, the interaction of pyrrolizidine alkaloids with ornithine delta-aminotransferase seems like not related to its antimalarial property; therefore, there should be other proteins/genes expressed by *P. falciparum* abled to be targeted in claiming the antimalarial agent. In the antidiabetic activity, the hypoglycemic effect of *Ageratum conyzoides* could be due to the presence of chromene compounds. In the *in silico* prediction, precocene I, precocene II, and VMDC show lower binding energy than other compounds in *Ageratum*. The activity of goat weed as an MMP-9 inhibitor could be due to the sterol compounds since this class exhibits relatively good binding energies in the *in silico* prediction. This activity could associate with its pharmacological activity as an anticancer. The correlation between *in silico* and the classical study is highlighted in Table 4.

In the topical pharmaceutical formulation aspect, Ageratum combined with other herbals has been well applied. The choice in the topical route could be due to some reasons. First, this herb is most likely indicated as a wound-healing agent either in the common wound or burned, antimicrobe, and arthrosis, by a topical route, and the drug would be interacting faster with the target site via local effect. Second, the topical route is chosen according to the toxicological effect of the liver, kidneys, and blood cells after long-term exposure as confirmed by both *in vivo* and *in vitro* assay. Last but not least, the unpleasant smell of goat weed could be the reason why the nonsystemic route such as topical is preferable for its pharmaceutical dosage form. The gel formulation is suitable for its indication in burns healing process due to its water base preparation giving cool and comfortable feeling during application. Moreover, the water-based gel may easily be absorbed by the wound dissolving like an interface between the gel and the wound excretes.

## 7. Conclusion

Based on the review, *Ageratum conyzoides* L. has a great deal of potency and clinical effect which is beneficial. Further efforts should be continued to decide the toxicity and adverse side effect of this herbal extract in the clinical study because there have not studied the specific toxicity as well as its adverse side effect. To date, there has not been much development in the formulation of this herb by using more advanced technology which can increase the economic value of this plant. Therefore, this could be a good innovation in the development of dosage form formulation utilizing *Ageratum conyzoides* L.

## Figures and Tables

**Table 1:** Seven compounds which would be predicted to interact molecularly with the respective protein target leading to their drug effect to the corresponding diseases.

Ligands	Protein target	Disease target
Precocene I	Cyclooxygenase-2	Pain
	MMP-9	Rheumatoid arthritis

Precocene II	Cyclooxygenase-2	Pain
	MMP-9	Rheumatoid arthritis

VMDC	Cyclooxygenase-2	Pain
	MMP-9	Rheumatoid arthritis

Beta-sitosterol	Tyrosinase	Antioxidant
	Glycoside hydrolase	Hyperglycemic
	4-Diphosphodicytidyl-2-C-methylerythritol	Antibacteria

Stigmasterol	Human p53, MMP-9	Cancer
	Tyrosinase	Antioxidant
	Glycoside hydrolase	Hyperglycemic
	4-Diphosphodicytidyl-2-C-methylerythritol	Antibacteria

5-Methoxynobiletin	MMP-9	Rheumatoid arthritis
	Ornithine delta-aminotransferase	Antimalaria

Lycopsamine	Ornithine delta-aminotransferase	Antimalaria

**Table 2:** Docking results performing binding energy of ligands from *Ageratum conyzoides* interacting with seven proteins playing a role in diverse pathologic condition.

Ligands	Binding energies (kcal/mol)						
	5m8s	3lg0	4cox	1i52	2zq0	4ibu	1l6j
Precocene I	−7	−7.2	−7.7	−6.2	−6.4	−4.2	−5.1
Precocene II	−6.8	−7	−7.6	−6	−6	−4.3	−5.3
VMDC	−7.4	−8	−7.5	−6.3	−6.1	−4.6	−5.3
Beta-sitosterol	1.8	−8.3	−8.9	−6.6	−2.5	−2.7	−6.9
Stigmasterol	−2.8	−8.4	−7.4	−6.8	−4	−3	−6.6
5-Methoxynobiletin	−4.7	−5.9	−8.5	−7	−3.5	−1.5	−6.2
Lycopsamine	−5.6	−4.5	−5.8	−5.1	−6.1	−4.3	−5.1

**Table 3:** Types of ageratum extract formulated in pharmaceutical dosage form, the respective active ingredients, and the protein target being studied.

Extract		Formulation	Active ingredient	Protein target
Part	Solvent			
Root	Ethanol	Ointment	Alkaloids	Ornithine delta-aminotransferase

Aerial part	Methanol	Emulsifiable	Precocene I	Cyclooxygenase-2, MMP-9
		Concentrate	Precocene II	

Aerial part	Dichloromethane	Emulsifiable	Precocene I	Cyclooxygenase-2, MMP-9
		Concentrate	Precocene II	

Aerial part	Hexane	Emulsifiable concentrate	Precocene I	Cyclooxygenase-2, MMP-9
			Precocene II	

Leaves	Ethanol	Gel	Alkaloids, flavonoids	Ornithine delta-aminotransferase, MMP-9

Aerial part	Ethanol	Nanoemulgel	Alkaloids, flavonoids	Ornithine delta-aminotransferase, MMP-9

**Table 4:** Correlation between *in silico* and the classical study of seven compounds identified in *Ageratum* in which most of compounds have a positive correlation except for lycopsamine.

Compounds	*In silico* methods	Classical methods	Therapy
Precocene I	+	+	Pain management, antidiabetes
Precocene II	+	+	Pain management, antidiabetes
VMDC	+	+	Pain management, antidiabetes
Beta-sitosterol	+	+	Anticancer
Stigmasterol	+	+	Anticancer
5-Methoxynobiletin	+	+	Antibacterial agent
Lycopsamine	−	+	Antimalaria

## Data Availability

This is a review article; hence, there is no data availability applied by the authors.

## Supplementary Materials

Supplementary Materials S1. The procedure of molecular docking using AutoDock Vina Program.
