# Supplementary material for: Medicinal Effect, In Silico Bioactivity Prediction, and Pharmaceutical Formulation of Ageratum conyzoides L.: A Review
Source: Scientifica (Cairo). 2020 Oct 13;2020:6420909. doi: 10.1155/2020/6420909 (PMC7578719; doi:10.1155/2020/6420909)
Supplement: Supplementary Materials — S1. The procedure of molecular docking using AutoDock Vina Program. [file 6420909.f1.docx]

# Supplementary Materials

# Medicinal Effect, *In Silico* Bioactivity Prediction and Pharmaceutical Formulation of *Ageratum conyzoides* L.: A Review

**S1. The procedure of molecular docking using AutoDock Vina Program**

**Software and hardware**

All 3D structure of the proteins was downloaded from the Protein Databank (PDB, [www.rcsb.org](http://www.rcsb.org)), and the structures of all lignads were collected from PUBCHEM <https://pubchem.ncbi.nlm.nih.gov/>). The virtual screening used molecular docking protocol in AutoDock Vina ([autodock.sripps.edu](http://www.sripps.edu)) and the output was visualised using Discovery Studio 3.5 ([www.accelrys.com](http://www.accelrys.com)). HP laptop with Core i3 processor on Windows 10 operating system with 4 GB RAM and 500 GB Hard Disk was the hardware.

**Method**

The proteins were prepared using AutodockTools 1.5.6 [1] whereby polar hydrogens were retained and the molecule was assigned with Kollman charges. The grid box was automatically defined by PyRx program (exhaustiveness = 8; size 25, 25, 25 and and the centre of coordinate is according the individual protein structures) and the docking was run using AutoDock Vina embedded in PyRx program [2]. The output was then collected as csv file and the compounds were tabulated according to the free energy of binding. The pose of docking was then visualized using Biovia Discovery Studio 2016.

**References**

[1] G. M. Morris, H. Ruth, W. Lindstrom, M. F. Sanner, R. K. Belew, D. S. Goodsell, A. J. Olson “Software news and updates AutoDock4 and AutoDockTools4: Automated docking with selective receptor flexibility,” *Journal of Computational Chemistry*, vol. 30, pp. 2785-2791, 2009.

[2] O. Trott, A. J. Olson, , “Software news and update AutoDock Vina: Improving the speed and accuracy of docking with a new scoring function, efficient optimization, and multithreading,” *Journal of Computational Chemistry*, vol. 31, pp. 455-461, 2010.
